# Mucin-Based Biomimetic Patches for Dynamic Heart Repair: Auxetic Structures and Translational Challenges

**DOI:** 10.1007/s13239-026-00824-2

**Published:** 2026-02-26

**Authors:** Rishatani Gunasegaran, Rania Hussien Al-Ashwal, Norhana Jusoh, Muhammad Hanif Ramlee, Sadeq M. Al-Hazmy

**Affiliations:** 1 Department of Biomedical Engineering & Health Sciences, Faculty of Electrical Engineering, Universiti Technologi Malaysia, 81310 Johor Bahru, Johor Malaysia; 2 Advanced Diagnostic and Progressive Human Care Research Group (Diagnostic), Health and wellness Research Alliance, Universiti Technologi Malaysia, 81310 Johor Bahru, Johor Malaysia; 3 Medical Devices and Technology Centre (MEDiTEC), Institute Human Centered Engineering, Universiti Technologi Malaysia, 81310 UTM Johor Bahru, Johor Malaysia; 4 Rehabilitation Nexus Research Group (RehabNex), Universiti Technologi Malaysia, 81310 UTM Johor Bahru, Johor Malaysia; 5 Department of Chemistry, College of Science, Qassim University, 51452 Buraidah, Saudi Arabia

**Keywords:** Biomimetic patch, Bioadhesives, Heart, Auxetic structure, Multilayered Architecture, Interlocking, Crosslink

## Abstract

**Purpose:**

This scoping review examines strategies to improve adhesion and integration of epicardial patches within the dynamic and wet cardiac environment. It emphasizes mucin-based bioadhesives and auxetic designs as potential solutions for long-term tissue-conformal myocardial repair.

**Methods:**

Approximately 150 peer-reviewed articles published between 2010 and 2024 were identified through a structured literature search conducted across PubMed, Scopus, and Web of Science. The studies cover adhesive biomaterials, structural patch designs, and epicardial tissue repair approaches were screened and thematically synthesized to map current advances, limitations, and translational gaps.

**Results:**

Commercially available epicardial patches fail to maintain adhesion under cyclic loading and fluid-rich conditions. Among bioadhesives, mucin demonstrates promising wet adhesion, viscoelasticity, and biocompatibility. Nonetheless, its translation is limited by batch-to-batch variability, purification challenges, and potential immunogenicity. Dopamine-, hyaluronic acid-, and alginate-based adhesives provide alternatives but remain constrained by oxidative instability, limitted long-term durability, or inadequate mechanical performance. Structural innovations such as auxetic geometries improve patch flexibility, strain distribution, and conformability compared with multilayered or microneedle-based strategies. However, challenges related to fabrication, sterilization, scalability and potential interference of cardiac anatomy andphysiology require further investigation.

**Conclusions:**

The synergistic integration of mucin-based adhesives with auxetic designs presents a compelling pathway toward durable, biocompatible, and tissue-conformal cardiac patches. Addressing manufacturing scalability, reproducibility, and immunological safety will be critical to advancing these concepts toward clinical applications.

## Introduction

Heart tissue undergoes continuous cyclic strain, with regional stresses reaching ~22 MN mm^−2^ and volumetric expansion of ~22% during contraction [1–4]. The circumferential fibre arrangement from the epicardium to the endocardium contributes to anisotropic mechanical responses necessary for coordinated cardiac motion [1–6]. At the microscopic level, the epicardium contains glycoproteins, proteoglycans, and structural proteins such as collagen types I/III, fibronectin, and laminin [6–14]. These molecules provide hydration, anchorage, and mechanical stability, supporting both native function and biomaterials interaction. Among extracellular components, hyaluronic acid contributes to hydration and viscoelasticity but exhibits weak intrinsic adhesion. Although chemical modification can enhance its adhesive potential, yet rapid in vivo degradation limits sustained performance on the epicardium [15–19]. This underlines the need for more robust adhesive systems tailored to dynamic wet cardiac environments. These macromolecules interact with functional groups on the patch through hydrogen bonding, ionic interactions, and, in some cases, covalent bonding. Integrins on epicardial cells play a crucial role in mediating cell–matrix adhesion and can bind to bioinspired motifs like RGD peptides incorporated into the patch design [20, 21]. Moreover, to enable seamless contraction, the heart is surrounded by a pericardial fluid cavity, which protects and lubricates the epicardial tissue. It contains lubricin, which regulates tissue adherence and reduces overall friction during contraction [22–25]. This protective mechanism create a persistently wet microenvironment that sustains hydration and regulates biomechanical processes at the cardiac surface.

Furthermore, at the mesoscopic level, extracellular matrix fibre alignment is arranged transmurally from the epicardium to the endocardium, with  a progressively increasing longitudinal component toward the endocardial layer, resulting in directional stiffness and anisotropy [26, 27]. The cardiac anisotropy is characterized by ratios ranging from 1.99 to 3.99 [1, 26–29], although some studies report 1.70 to 3.50 as relatively close to epicardial tissue [30]. Another important factor is Poisson’s ratio, which characterizes deformation and compressibility of tissue at the macroscopic level. The epicardial tissue extends in both directions, resulting in a negative Poisson’s ratio ranging from – 0.2 to – 0.5, reflecting the relationship  tbetween transverse and longitudinal strain under applied force [1, 29–32]. Young’s modulus is also significant, reflecting tissue stiffness, with values reaching approximately to 50 kPa in healthy hearts and up to 1 MPa in injured hearts tissue [1, 29–33].

The structural heterogeneity, physiological environment, and mechanical attributes of the organ should be considered during the fabrication of biomimetic patches for repair. These factors enable patches to complement native tissue, withstand physiological stress, and interact with cells to maintain structural integrity intact. An ideal biomimetic patch should mimic the natural microenvironment while sustaining durability and biocompatibility. Additionally, features such as microporosity, surface roughness, and hydrophilicity further enhance adhesion by increasing the contact area and facilitating mechanical interlocking and cell infiltration [34–36]. Altogether, effective patch integration at the microscopic level relies on both biochemical compatibility and physical interfacial design that supports stable bonding with the native epicardial environment.

These mechanical properties, continuous movements driven by muscle contractions, and the wet environment create challenges for biomimetic patches to replicate resilience and ensure robust adherence. Current patches often fail to match the heart’s compliance, leading to premature detachment or breakage that interrupts repair [1, 30, 37, 38]. Unlike static organs, the dynamic nature of heart tissue requires adhesive patches with attributes such as tissue binding, biocompatibility, viscoelasticity, volumetric deformation tolerance, and cyclic contraction resistance. However, current patches fail to achieve long-term adhesion.

Mucin-based adhesives show promise but face significant translational challenges: their biological origin introduces batch-to-batch variability, purification processes may alter glycosylation patterns critical for adhesion, and preparations derived from non-human sources can elicit immunogenic responses. These issues highlight the need for standardized production, careful immunological assessment, and scalable processing before reliable clinical translation [39–49].

In parallel with advances in adhesive materials, structural innovations in patch design have gained traction. Auxetic structures characterized by a negative Poisson’s ratio, demonstrate enhanced mechanical adaptability, enabling patches to conform to complex and dynamic tissue geometries without delamination [28, 30, 50, 51]. Compared with multilayered or microneedle-based strategies, auxetic designs offer superior strain distribution and mechanical stability under cyclic loading [52–55]. This review scrutinizes adhesive materials, with particular focus on mucin-based adhesives and auxetic structures, to identify strategies for achieving robust adhesion to the heart while matching the mechanical and physiological properties of native cardiac tissue.Given the overlapping chemistries and mechanical functions of these bioadhesives, materials are discussed comparatively across sections to highlight shared limitations and distinct translational challenges.

## Adhesive Materials for Epicardial Patches

Biomimetic patches are often incorporated with adhesive materials through methods such as layering, crosslinking, copolymerization, and surface coating, which interact with tissue to provide adherence to the heart. Adhesive substances either diffuse into tissue to form interfacial linkages with biomolecules or exploit differences in surface charges and electronegativity to initiate adhesive forces [56, 57]. Currently, natural and synthetic adhesives are widely used in biomimetic patches due to their elasticity and ability to withstand pulsatile movement [58].

**Fig 1.**
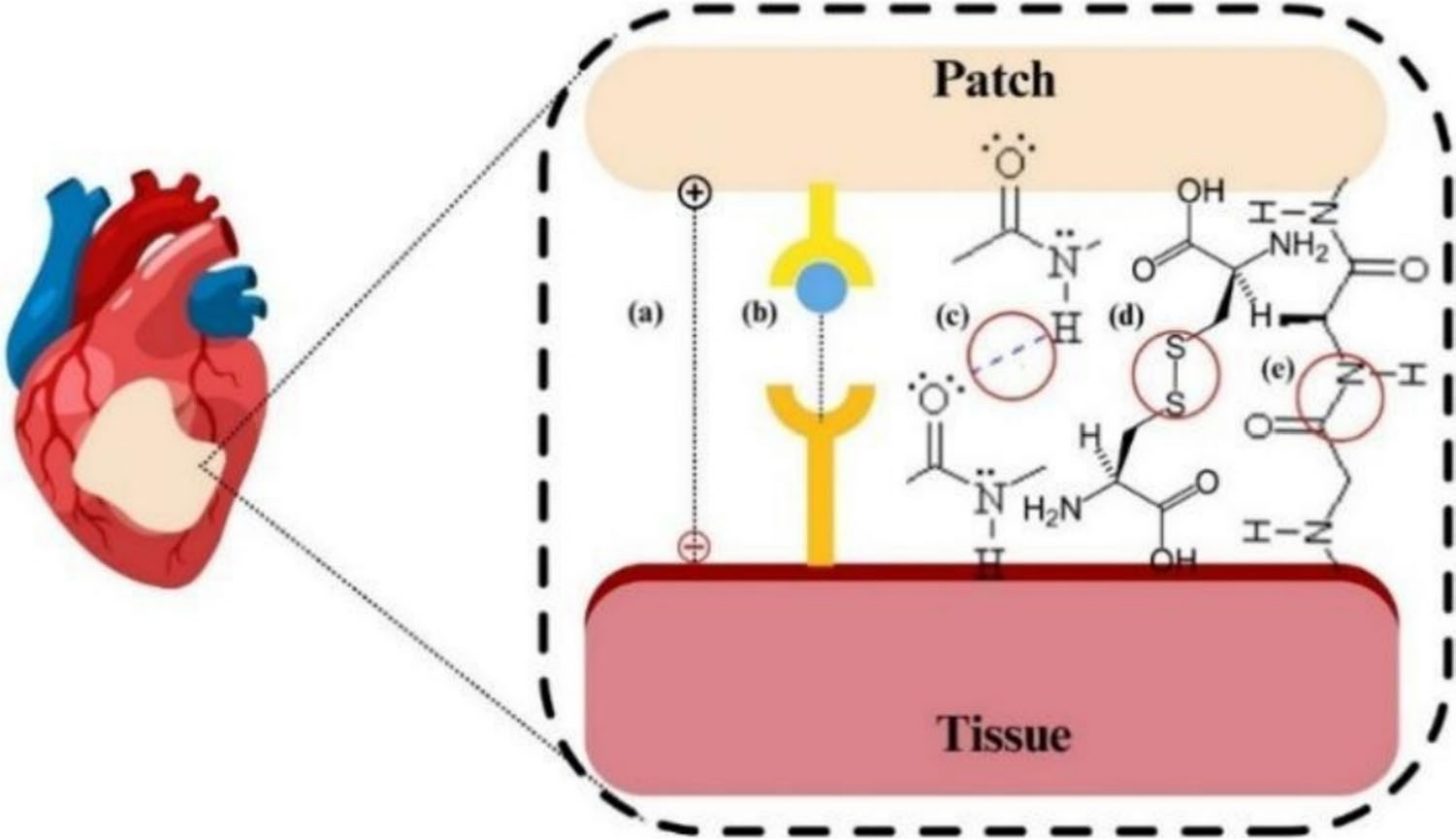
Various bonds formed between tissue and adhesive patches; **a** Electrostatic interactions, **b**Van der Waals interactions; **c** Hydrogen bonding; **d** Disulfide bonds; **e** Amine bonds

Natural adhesives derived from proteins and polysaccharides, including hyaluronic acid, dopamine, and alginates-based systems, are valued for their biocompatibility, sustainability, and intrinsic adhesive properties [58–61]. Adhesion of a cardiac patch to epicardial tissue is mediated through covalent, ionic, hydrophobic, electrostatic, and hydrogen bonding interaction (Fig. 1) [45, 56–65]. Dopamine-based adhesives provide robust initial wet adhesion but are chemically unstable under oxidative conditions, potentially generating cytotoxic by-products that limit long-term safety [66, 67]. Hyaluronic acid contributes hydration and viscoelasticity but exhibits weak intrinsic adhesion and undergoes rapid in vivo degradation, necessiating chemical modification for cardiac applications [15–18]. Alginate hydrogels demonstrate moderate biocompatibility and are suitable for injectable systems, however, their low shear resistance and limited mechanical resilience limit long-term epicardial applications [68–73]. In addition to these specific cases, many natural adhesives exhibit favorable attributes such as viscoelasticity, diffusion capacity, and antimicrobial or anti-inflammatory activity, which may enhance adhesion and healing at the injury site [59, 60, 74–77].

### Mucin and Mucin-Mimetics

Animal protein–based adhesives represent a promising class of natural adhesives. Mucin and its derivatives are underexplored glycoproteins typically obtained from respiratory mucus, the gastrointestinal tracts of ruminants, or gastropods such as snails and slugs [39, 78, 79]. In animals, mucus secreted by epithelial linings functions as a protective barrier that provides lubrication and adhesion, whereas gastropods secrete mucus to glide across rough surfaces and reduce friction [39–45].

**Fig 2.**
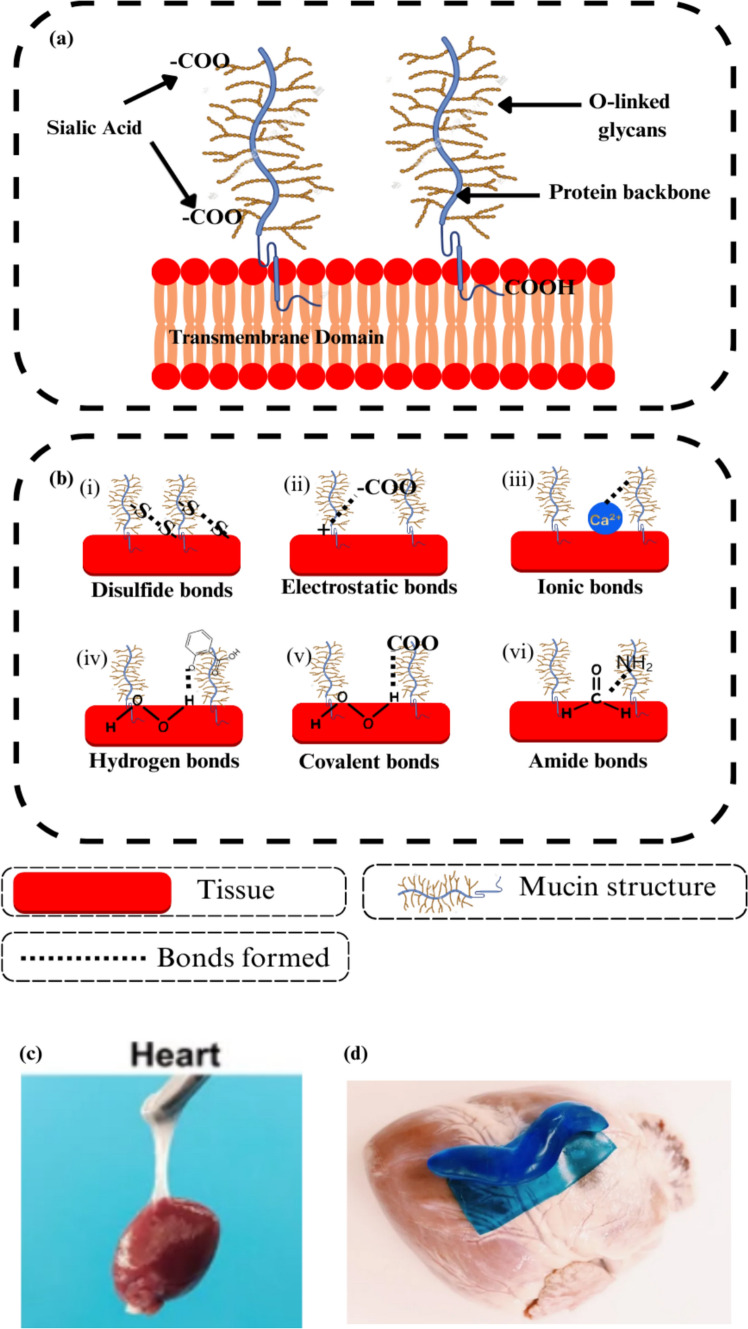
Mucin adhesives; **a** Molecular structure of mucin highlighting functional groups involved in intermolecular interactions. **b** Schematic representation of mucin–tissue adhesion, illustrating the formation of intermolecular bonds between mucin molecules and tissue surfaces that contribute to adhesive performance. **c** Application of mucin adhesives on the rat heart, Adapted from [46]. **d** Application of mucin adhesives to porcine heart tissue, Adapted from [90]

The adhesive properties of mucin arise from its distinctive molecular architecture comprising glycan side chains, protein backbone, negatively charged groups (sialic acid and sulfates residues), secondary metabolites, a polyanionic central region with hydrophobicand hydrophilic domains, Ca^2+^ ions, and disulfide bridges (Fig. 2) [80–82]. Glycoproteins primarily contribute to hydrogel formation, while smaller peptides, hydrophobic lipids, lectins, metal ions, sulfonated molecules, and aromatic amino acids influence adhesive performance [82, 83].

Functional groups such as carboxyl, hydroxyl, and sialic acid residues mediate hydrogen bonding with collagen and electrostatic interactions with laminin and fibronectin. These amphiphilic interactions also extend to materials such as chitosan and polyethylene glycol (PEG), forming hydrogen, ionic, π–cation, and hydrophobic interactions [45, 81–85]. Recent advances have also explored mucin mimetics and natural mucin-based hydrogels in epicardial and wound applications, reinforcing their potential as scalable bioadhesives. Crosslinking with PEG derivatives or acrylate functional groups further enables covalent network formation, producing stable polymeric matrices that adhere strongly to tissues [40, 85, 86]. Mucin’s carbohydrate domains interact with lectins in slightly acidic environments, thereby supporting cell adhesion and tissue repair [87, 88]. Reports indicate favorable cytocompatibility of mucin-based scaffolds without notable toxicity in relevant biological settings [40, 85].

Physical and chemical properties, including reversible gelation, viscoelastic behavior, shape recovery, and thiol-rich domains further contribute to mucin’s robust wet adhesion performance[40]. Snail mucin, rich in sulfated glycosaminoglycans, aromatic amino acids, and divalent cations, has demonstrated exceptional adhesion strength (Fig 2c), outperforming fibrin glue in term of interfacial toughness [89, 90].

Despite these advantages, mucin faces translational challenges: its biological origin introduces batch-to-batch variability, purification can alter glycosylation patterns critical for adhesion, and certain preparations, particularly from non-human sources, may trigger immunogenic responses. Addressing these barriers requires standardized production pipelines, scalable purification techniques, and rigorous immunological evaluation before mucin-based adhesives can be advanced into clinical epicardial applications [39–49]. Altogether, mucin combines excellent biocompatibility, tunable viscoelasticity, and strong adhesive capacity, making it highly attractive for biomedical applications.

In another study, snail-glycosaminoglycan was incorporated into  GelMA to achieving strong tissue adhesion up to 70 kPa, penetrating tissue and withstanding continuous bending and twisting motions [89]. The hydrogel-based patch formed amide bonds between carboxylic acid and amine groups in tissue, initiating hydrogen and ionic bonds. These patches exhibited elastic behaviour with concentration-dependent flexibility for soft tissue interaction. Furthermore, mucus from the mollusc *Arion subfusc* contains zwitterionic moieties that intertwine to form double-network hydrogels with exceptional toughness as shown in Fig.2d. The bilayer adhesive structure, comprising adhesive and dissipative surfaces, prevents undesired adhesion to adjacent tissues and achieves adhesion energies of ~ 1000 J m^−⁻2^, surpassing commercially available adhesives [90].

Mucin-derived hydrogels have also been fabricated, showing strong tissue adhesion via molecular bonds such as catechol–thiol linkages, tenable gelation, and water-tolerant cohesive properties [47].

In a study, slug mucus was used as a suture replacement, exhibiting exceptional wet adhesion and tailorable mechanical properties. Its biochemical composition includes primary metabolites (carboxylic acids, fatty acyls, organooxygen compounds, pyridines, phenols, indoles) and secondary metabolites (amino acids, peptides, fatty acids, carbohydrate derivatives, pyridine carboxylic acids, benzenediols) [48]. Additionally, mucus contains diverse metal ions such as copper (Cu), iron (Fe), manganese (Mn), and zinc (Zn), which contribute electrostatic and cationic interactions that reinforce tissue adhesion [49].

Owing to its unique molecular structure and diverse bioactive components, slug mucus can achieve quick, prolonged, and robust adhesion without further chemical modification, outperforming many natural and synthetic adhesives in epicardial applications.

### Mussel Protein-based Adhesives

Marine adhesive proteins (MAPs) are bioadhesive proteins that are secreted by marine mussels. They are potent adhesives which can adhere to various surfaces by the ability to form catechol-based interactions even under wet environments and can cure rapidly [16]. MAPs are also suitable to be used in biomimetic patches to attach dynamic organs and excellent wet adhesion. The MAP is composed of catechol-based side chains 3,4-dihydroxy-l-phenylalanine (DOPA), which are able to interact chemically and physically with thiols, amines and aldehydes that are present on the tissue [91]. Not only that, but the side chains have also been able to form electrostatic interactions with oppositely charged molecules and hydrogen bonds. The DOPA can react with quinone to generate DOPA-quinone complex extending bonds with thiols and amines enhancing cohesion, while with the hydroxyl groups strong hydrogen bonds will be formed on the surface of the biological tissue [92, 93]. Moreover, through metal chelation DOPA initiates bonds with the free moving ions present on the surface of the tissue while the aromatic ring is involved in π–π stacking with aromatic molecules in the tissue [68, 94]. Charged residues in MAPs (like lysine or arginine) interact with oppositely charged moieties to initiate electrostatic attraction and the hydrophobic groups that improve adhesion by removing water molecules at the interface [95].

In a study conducted, MAP is fused with silk fibroin (SF) and used as an adhesive for the fabrication of double layered cardiac patch to promote regeneration at the infarcted heart [96]. The adhesive strength of the patch is compared with the commercially available medical grade glue, cyanoacrylate using a porcine epicardium. The adhesive strength was recorded at 101.64 ± 4.01 kPa which is higher than the SF patch. In the dynamic beating heart, attachment of the patch sustained for more than 2 weeks due to a stronger formation of amine-aldehyde complexes. However, a high degradation rate was observed, where only 30% of the patch remained attached to the patch as the adequate regeneration of the heart requires a time span of 4 weeks. In another study, an adhesive protein based immiscible condensed liquid system is made using MAP and hyaluronic acid to be attached to the scarred epicardium to enhance the repair. These complexes extended electrostatic interactions with tissue structure and allow robust adhesion remaining stable on the surface [95].

Although these formulations demonstrate improved adhesion compared with conventional materials, hyaluronic acid itself remains limited by weak intrinsic adhesion, rapid degradation, and the need for chemical modification to maintain stability under dynamic cardiac conditions. Excessive modification can also introduce cytotoxic effects, further complicating clinical translation. Consequently, while HA-based systems show promise, their long-term performance and reproducibility remain major challenges for sustained epicardial repair.

Dopamine (DA) and its derivatives are widely studied adhesive materials for cardiac patches. Dopamine (DA) is a small molecule with catechol and amine groups, structurally analogous to mussel adhesive proteins [97, 98]. Adhesion is facilitated through dopamine–dopamine covalent bonds formed via quinone moieties [99]. In alkaline conditions, DA polymerizes into polydopamine, which is generally non-toxic to cells [66, 100]. DA also interacts with diamines, carboxyl groups, hydroxyls groups, and quinone groups through as-Michael addition, hydrogen bonding, or covalent crosslinks during polymerization [92, 101].

Despite these advantages, dopamine-based adhesives face critical limitations. Their oxidative instability leads to excessive catechol oxidation, generating reactive oxygen species (ROS) and cytotoxic by-products that place stress on surrounding cells [67, 102–105]. They also suffer from variable gelation times, poor network stability, and limited durability, reducing their long-term reliability [67, 105]. Consequently, while dopamine provides strong short-term adhesion, its cytotoxicity and poor stability necessitate chemical modification or combination with other biomaterials for safe and clinically viable epicardial applications.

Experimental efforts highlight both potential and limitations. In a study, dopamine–gelatin (GelDA) conjugates combined with dopamine-modified polypyrene (Ppy) demonstrated wet adhesion in the presence of Fe^3+^ ions via chelation [106].

GelDA improved dispersion and adhesion, but high Ppy content produced long gelation times, poor stability, and lower adhesive strength on heart tissue compared to skin. In another approach, dopamine with hyperbranched polymers, polymerized via Fe^3+^, created a paintable cardiac patch that adhered for four weeks without detachment [99]. However, excessive adhesive strength damaged myocardial tissue, and gelation exceeding 30 s reduced surface wettability.

Overall, dopamine-based adhesives offer strong initial adhesion but remain constrained by oxidative instability, cytotoxic by-products, and poor durability, reinforcing the need for further chemical modifications or hybrid designs for clinical translation. Hyaluronic acid (HA) is a naturally occurring polysaccharide widely applied in tissue engineering [15]. In its native form, HA exhibits poor mechanical strength and weak adhesion due to its hydrophilic nature and high-water content, leading to rapid in vivo degradation [16]. To overcome these limitations, HA is often chemically modified with catechol, proteins, methacrylates, thiol groups, surfactants, or other polysaccharides [17, 18, 107]. These modifications alter its physical and mechanical properties, enabling additional covalent interactions such as disulfide bonds, Schiff bases, and π–π stacking beyond its typical hydrogen and electrostatic bonding [108, 109].

Several engineered HA-based systems demonstrate improved cardiac performance. A dual therapy using aldehyde-modified HA (HA-ALD) sponges carrying nitric oxide achieved adhesion strengths of 17.64–57.17 kPa, though high aldehyde content induced cytotoxicity, limiting the usable formulation to 2% HA-ALD, which remained unstable [110]. In research, the author developed hydrogel “tissue tape” using catechol-conjugated (HA-CA) and pyrogallol-conjugated HA (HA-PG), achieving four-fold and two-fold stronger adhesion than unmodified HA, respectively. However, these systems were not tested in vivo on dynamically beating hearts [111]. Another approach combined oxidized sodium HA (HA-CHO) with Schiff base chemistry, further enhancing tissue adhesion [112].

While chemical modification enhances HA’s adhesive and mechanical properties, it also introduces challenges such as cytotoxicity (aldehyde, catechol), variability in modification processes, and concerns over reproducibility. Consequently, although modified HA formulations show promise, the reliance on chemical functionalization complicates their durability and clinical translation.

### Alginate and Other Biopolymers

Alginate is one of the plants-derived from anionic polysaccharide that is widely used in tissue engineering [68]. It is often processed into hydrogels due to its hydrophilicity and swelling ability. Alginate-based hydrogel have received considerable  attention as an adhesive due to its biocompatibility, gel-forming structure which facilitate attachment the wet and dynamic organs. However, alginate is relatively a bioinert polymer. Thus, to resolve this issue, alginate is modified with conjugates or undergoes ionic crosslinking with Ca^2+^ ions [69]. Applying this perspective, alginate-based hydrogels are often introduced with catechol moieties to produce mussel protein like structure which has a tailorable adhesion [70]. Not only that, it is often combined with other polymers like chitosan and gelatin to initiate inter polyelectrolyte complexes where the breakage and binding of the negatively charged molecule to the chitosan to activate bonds formation and reaction in the alginate [71, 113].Furthermore, alginate is often functionalized with catechol, aldehyde, or thiol groups to form covalent bonding and enhanced intermolecularinteractions.

Catechol-modified alginate hydrogels have been utilized as adhesives for an epicardial patch [96]. The patch has been embedded with biosensors and adhered to the surface of the myocardium to allow the early detection of various cardiac conditions. The patches showed adequate adhesion in the dynamic heart conditions when attached to the *in vivo* models. Moreover, the researcher mentioned that the fibrous structure of the patch itself helps in withstanding the mechanical force of the dynamic heart. Similarly, a biomimetic patch incorporating pressuresensitive transistors to sense the cardiac beating [114] Catechol modified alginate is used to provide robust adhesion to withstand the pulsatile motion of the heart. It was found that the alginate based adhesive acts as a cover and does not interfere with the heart surface or damaging  the embedded electrodes. Furthermore, the modification of alginate hydrogels enhanced overall mechanical stability and adhesion under  cyclic cardiac contraction.

### Mucin Based Adhesives vs. Other

Among bioadhesive materials, mucus-based systems stand out as the most effective and versatile in wet biological environments. Rich in interactive groups such as carboxyl, hydroxyl, sialic acid residues, sulfates, amines, and glucosamines, mucin forms hydration layers and dynamic supramolecular bonds that sustain adhesion under cyclic strain [46]. Reported adhesion strengths of mucin-based adhesives (30–100 kPa) surpass those of dopamine-based systems (10–22 kPa) [97], hyaluronic acid formulations (17.64–57.17 kPa) [110], and alginate (~7 kPa) [113], and are comparable to mussel protein adhesives (23.38–101.64 kPa) [96]. However, adhesion values reported across studies were obtained using different testing modalities (e.g., lap shear, peel, tensile pull-off), and direct numerical comparison should therefore be interpreted cautiously. Importantly, these values fall within or exceed epicardial stresses of 10–50 kPa during cardiac cycles, suggesting sufficient interfacial stability without excessive strain [115].

Despite this superior balance of wet adhesion and biocompatibility, mucin-based adhesives face translational barriers including variability, purification challenges, and potential immunogenicity. In comparison, dopamine adhesives suffer from oxidative instability and cytotoxic by-products; hyaluronic acid requires extensive modification yet degrades rapidly; and alginate, while biocompatible, shows poor shear resilience and is more suited as an injectable carrier. Collectively, mucin offers the strongest combination of stability and biocompatibility, but overcoming these material-specific limitations will be critical for durable clinical translation.

Furthermore, mucus adhesives demonstrate excellent toughness and mechano-stability, enabling them to cope with the dynamic environment of the heart. Through reversible interactions, mucin forms hydrogel networks that provide flexible adhesion on the epicardial surface without restricting cardiac motion [41, 90] Under cyclic shear conditions, as in a beating heart, mucus networks can rearrange up to100-fold faster, maintaining structural integrity while dissipating energy [115, 116] This contrasts with dopamine- and HA-based adhesives, whose covalent or catechol networks are canexhibit brittlness and prone to degradation. Consequently, mucin adhesives exhibit superior toughness and viscoelastic stability, distributing force evenly and reducing interfacial stress concentrations during contraction, whereas MAP and dopamine adhesivesdisplay more rigidly.

While mussel proteins and dopamine offer strong covalent bonding through catechol/DOPA groups, they often require chemical modification or catalysts. In contrast, mucin adhesives adhere strongly to wet tissue without additional crosslinkers, preserving biocompatibility and biodegradability [117]. They also gelates rapidly (as fast as 30 s) [85], compared with alginate (12–22 min), HA, mussel proteins, or certain reducing adhesive loss in pericardial fluid and improving fixation on the epicardium.

Overall, across parameters such as wet adhesion, mechanical resilience, and biocompatibility, mucus-based adhesives outperform other natural adhesives, represent one of the most promising choice for next -generation epicardial applications. The following table summarizes the strengths and limitations of each adhesive, highlighting their alignment with specific clinical requirements. The summary is shown in Table 1.

**Table 1:** Summary of key properties of mucin based and conventional adhesives, highlighting their adhesive strengths, viscoelasticity, mechano-stability and other properties

Adhesive Materials / Properties	Mucus adhesive	Dopamine	Hyaluronic acid	Mussel Protein	Alginate
Interactive compounds	Carboxyl, hydroxyl and sialic acid groups, sulphates, amines, cations and anions, glucosamines	3,4-dihydroxyphenylalanine (DOPA), catechol groups, amine groups, quinone groups	Hydroxyl groups, glucosamine, glycosidic groups	3,4-dihydroxyphenylalanine (DOPA), amine groups, catechol groups	Divalent cations, hydroxyl groups, carboxyl groups, phenols
Wet Adhesion	Yes	Yes	Slightly, needs modification	Yes	Slightly, needs modification
Adhesion strength (kPa)	30–100	10–22, varies with composition	17.64–57.17	23.38–101.64, varies with mixed polymer	~ 7, poor adhesion strength
Biocompatibility and Cytotoxicity	Enhances cell–cell interaction and proliferation. Limitations: batch-to-batch variability, purification difficulties, risk of immunogenicity.	Cytotoxicity depends on formulation. Unstable under oxidation, can generate cytotoxic by-products	Enhances cell proliferation. Weak adhesion, rapid degradation, requires chemical modification for stability	Can produce harsh residues at higher composition	Requires purification for good biocompatibility. Poor shear resilience, mostly used in injectable form
Mechano-stability	Excellent toughness	Poor network stability	Poor network stability due to rapid degradation	Decreased tensile strength	High fatigue resistance. Limited in load-bearing capacity under cyclic stress
Viscoelasticity	Maintains viscoelasticity under high shear	No	Viscoelastic only at higher concentration or temperature	Viscoelastic but brittle under stress	Only if ionic crosslinking applied
Gelation time	Starts from 30 s	Longer gelation time	Rapid gelation (~5 s with catalysts)	Longer gelation time (minutes–hours)	12–22 minutes
Reference	[41, 46, 85, 89]	[67]	[110]	[96]	[73, 113]

## Structural Design Strategies

Biomimetic patches attached to host tissue are subjected to significant mechanical loads from cyclic contraction and relaxation of the heart [30, 118]. These continuous movements challenge adhesion and can lead to patch detachment, permanent deformation, or tissue damage [118, 119]. Recent advancements in patch fabrication highlight the importance of both adhesive materials and structural design in achieving stable integration under dynamic, wet conditions.

Auxetic structures, characterized by a negative Poisson’s ratio, enhance conformability and stress distribution, enabling patches to adapt to repetitive deformation with reduced risk of detachment [1, 120–122]. Microneedle-based designs provide mechanical anchoring through controlled penetrating the epicardium and promoting physical interlocking [96, 123, 124]. Multilayered architectures further improve performance by separating functional roles across layers, thereby maximizing adhesion and compliance [96, 125–127]. Collectively, these design strategies work synergistically with adhesive materials to improve structural stability, increase tissue contact area, and maintain prolonged adhesion. In this review, interfacial adhesion refers to the physicochemical bonding strength at the patch–tissue interface measured under static or quasi-static conditions. In contrast, mechanical retention describes the ability of a patch to remain attached under cyclic cardiac motion through structural interlocking, penetration, or geometry-induced constraint.

### Auxetic Geometries

Auxetic structures are characterized by the unique property of expanding longitudinally when stretched laterally. They are composed of repeated cell units with inward-leaning walls that rotate during deformation, causing adjacent cells to move apart and creating expansion under tension. This bidirectional deformation results in a negative Poisson’s ratio, which arises primarily from the specific microstructure of the material rather than its chemical composition [28, 120]. Computational and experimental studies from 2022–2025 have confirmed that auxetic designs improve mechanical compliance under cyclic cardiac loading, conforming their translational relevance [121, 128–132].

**Fig 3.**
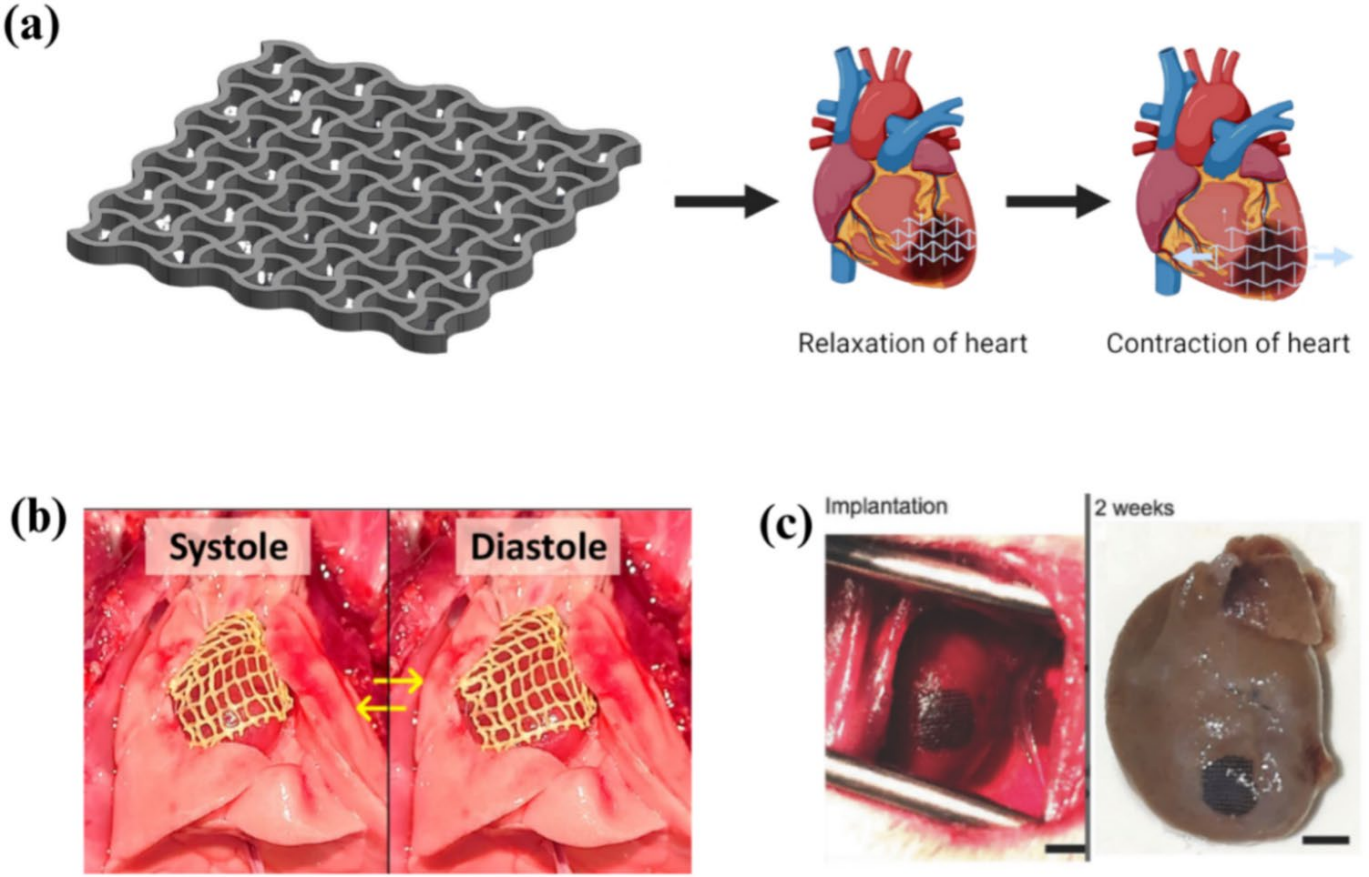
Auxetic geometries; **a** A schematic illustration of re-entrant auxetic structure displaying bidirectional expansion during heart contraction; **b** Application of adhesive auxetic geometries used in cardiac patch, Adapted from [121]; **c** Adhesive auxetic cardiac patch maintaining stable adhesion in a rat heart for 2 weeks, Adapted from [28]

Common auxetic mechanisms include re-entrant geometries, rotating units, and other specialized patterns that enable lateral expansion. Among these, re-entrant auxetic structures are considered  the most flexible and adaptable, characterized by inward-angled cell walls that allow bidirectional deformation. As illustrated in Fig 3a, they distribute mechanical strain evenly by expanding in both directions, conform to complex curved surfaces, and maintain stable contact under dynamic loading, offer a compelling platform  for soft tissue applications such as cardiac patches. In addition, re-entrant auxetic structures demonstrate excellent durability, shock absorption, energy transmission, and versatile design possibilities [28, 132–134].

A previous study[121] highlighted that auxetic lattice architectures enable bidirectional deformation and homogeneous stress distribution under cyclic loading, which are critical for maintaining stable contact on the beating heart as shown in Fig. 3b Additionally, but the auxetic geometries   have also been reported to maintain adhesion for up to weeks as their flexible structure reduce premature detachment, as shown in Fig 3c [28] Although the reported systems do not incorporate mucin directly, these geometries provide promising structural platform for integration with mucin-based adhesive layers. Through hydration-mediated bonding and viscoelastic energy dissipation, mucin is expected to further enhance wet-tissue adhesion and resistance to interfacial delamination during repetitive cardiac motion.

**Fig 4.**
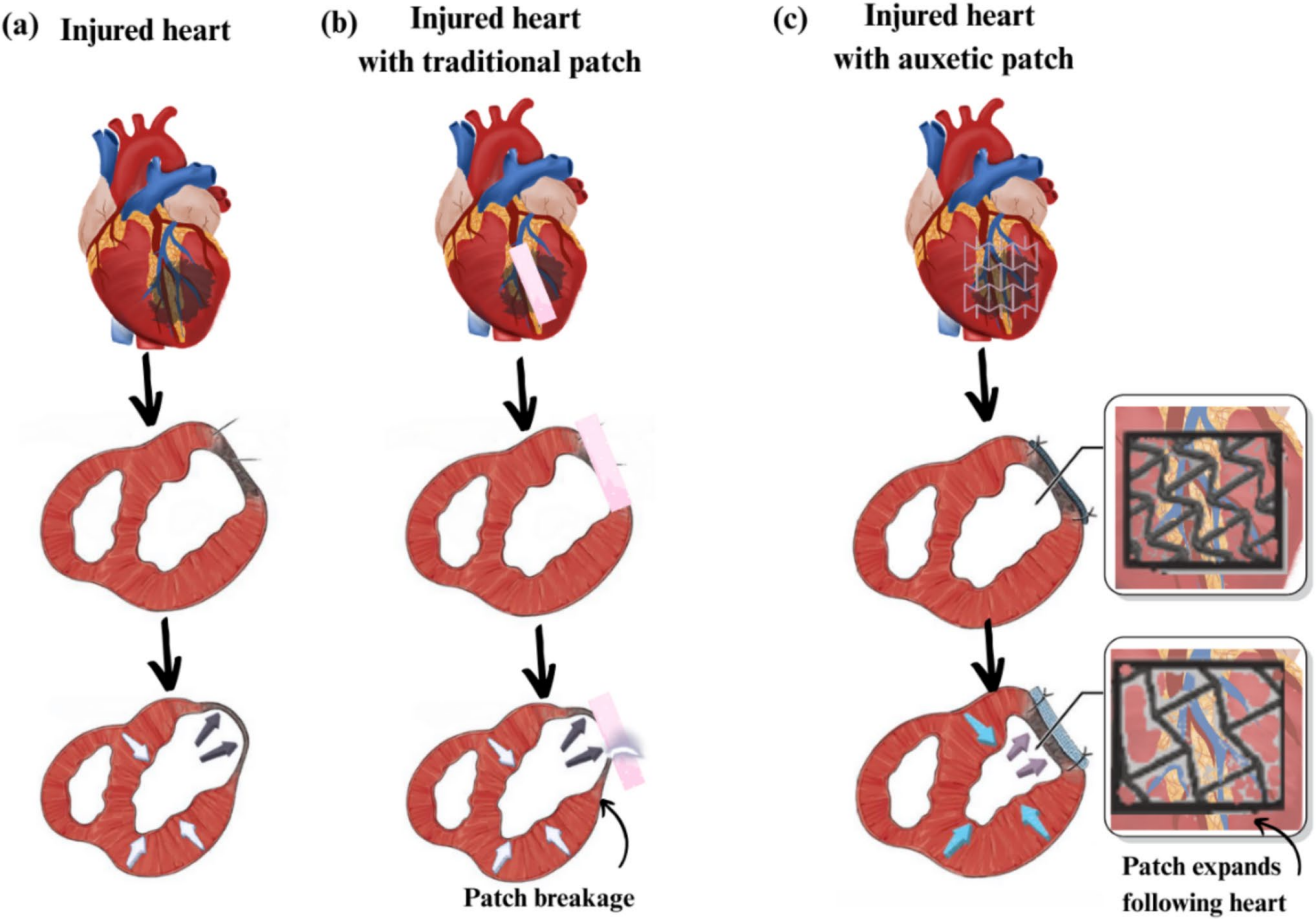
Attachment of patch on the injured region and anterior cross section showing force distribution; **a** injured region without patch showing bulging; **b** injured region with traditional patch showing breakage during systole; **c** injured region with auxetic patch showing expansion during systole

Another study reported that auxetic structures provide adequate mechanical support to the heart without compromising structural stability and may reduce premature patch detachment caused by stresses within the cardiac wall [1, 135]. The dynamic myocardium exhibits variable anisotropy and undergoes repetitive volumetric expansion and contraction, creating a fatigue loading environment that predisposes conventional patches to mechanical failure or detachment. By contrast, patches incorporating auxetic geometries expand laterally under tensile loading, enabling them to conform synchronously with the multidirectional deformation of the beating heart, as illustrated in Fig. 4.

Auxetic geometries mimic aspects of native epicardial mechanics, which have been reported to display slight auxetic responses under physiological strain. During repeated systole and diastole, auxetic patches distribute loads evenly across the tissue–patch interface, reducing localized strain concentrations that can trigger adhesive failure. This homogeneous stress distribution prevents crack initiation and edge lifting, two major causes of premature patch delamination under long-term cyclic loading [52, 136–138]. Both ex vivo and in vivo studies demonstrate that auxetic epicardial patches maintain close contact throughout the cardiac cycle, achieving improved mechanical integration compared with non-auxetic designs [31, 139, 140]. These properties make auxetic structures especially promising for cardiac applications, where materials must endure repetitive deformation while preserving stable contact with epicardial surfaces.

The geometrical flexibility of re-entrant auxetic patterns enables full-surface contact with the epicardium’s complex curvature, even under dynamic volumetric changes. Unlike conventional patches that stiffen or fail to accommodate surface deformations, auxetic patches exhibit superior synclastic adaptability, dynamically molding to the contracting and expanding heart [136, 141, 142]. Another study demonstrated that re-entrant honeycomb structures conform more effectively to curved surfaces and produce lower stress concentrations compared to non-auxetic structures, which tend to buckle under deformation [143] This adaptability minimizes localized stress at the patch–tissue interface and reduces risks of edge detachment or mechanical failure, which is especially critical under wet physiological conditions where shear forces can compromise adhesion.

In vivo models of dynamic organs support these findings. In lung models with large volume changes, auxetic patches preserved sealing and interface integrity more effectively than conventional designs. In cardiac studies, they outperformed fibrin-based adhesives in maintaining fixation under cyclic loading and adhesion strength reached up to 130 kPa [121]. By expanding laterally in synchrony with epicardial motion and dissipating shear stresses, re-entrant auxetic geometries offer the dual benefits of mechanical compliance and stable adhesion. Their properties, including high deformability, shear resistance, synclastic behavior, and tunable anisotropy, which can be tailored by adjusting unit-cell parameters such as re-entrant angle, strut dimensions, and cell size to optimize stability, auxetic response, and compatibility with cardiac mechanics [51, 136, 144–146].

In epicardial applications, re-entrant honeycomb auxetic structures were fabricated for myocardial infarction treatment, systematically varying unit-cell dimensions, strut length (320–480 µm), thickness (50–150 µm), width (240–360 µm), and re-entrant angle (40°–80°). These parameters significantly influenced the anisotropic ratio, Poisson’s ratio, and tensile strength of the patches. Using laser micro-ablation with chitosan and polyaniline, the fabricated auxetic patches exhibited anisotropy comparable to native cardiac tissue (1.9–3.9). However, ultra-thin microstructures displayed reduced mechanical strength, suggesting that larger-scale geometries may be required for durability [28].

Subsequent studies have explored alternative auxetic designs, including re-entrant honeycomb, sinusoidal ligaments, and lozenge trusses, to evaluate stiffness and Poisson’s ratio across dynamic organs. Length and width variations up to 2 mm, angles of 60°–70°, and thicknesses of 0.25–0.75 mm were tested, confirming that re-entrant structures provide the stiffness needed for cardiac applications, with computational models yielding reliable predictions [131, 135]. A recent study further demonstrated that varying volume fraction and thickness of auxetic patches allows tuning to match the dynamic properties of heart tissue [30]. Similarly, another study reported that a missing-rib design achieved favorable Young’s modulus, stiffness, and anisotropy, though its variable Poisson’s ratio produced greater expansion than expected [120].

Integrating mechanically adaptive auxetic geometries with robust bioadhesives such as mucin could yield epicardial patches that combine secure adhesion with compliance to dynamic cardiac motion. However, mucin-based systems still face practical barriers, including batch-to-batch variability, complex purification, and potential immunogenicity from non-human sources. Addressing these translational challenges remains essential for clinical implementation.

### Multilayer Architectures

Multilayer patches have been engineered to improve adhesion and mechanical strength by integrating distinct functional layers. For instance, a three-layer patch was designed comprising hydrophobic fluid and zwitterionic elastomer layers, which enhanced anti-fouling properties and prevented contamination [52]. In general, bilayer and three-layer designs are the most common, with each layer contributing specific functionalities to optimize adhesion and mechanical compliance [53, 147–149]. Importantly, multilayer architectures can be tuned to approximate the thickness of native tissue while maintaining adhesion up to 17.5 kPa [150], while layer-by-layer fabrication provides flexibility, enabling precise control over thickness and composition to adjust overall mechanical performance [53, 147–151].

### Microneedle-Based Patches

Microneedle patches achieve robust adhesion by penetrating tissue and creating interlocking interfaces, allowing stability even under cyclic deformation. Inspired by natural structures such as bee stings, they provide deeper penetration and stronger fixation without the need for external adhesives [152, 153] While microneedles are widely applied for therapeutic delivery, they have also been adapted to enhance epicardial patch retention by securing firm contact with dynamic tissues where adhesion strength up to 70 kPa were reported [55, 153–155]. Their microstructures, often modelled after insect stings, barbs, or feet, facilitate effective anchoring through mechanical integration with underlying myocardium [54, 55, 156].

To overcome the limited strength of soft microneedles, a backward-facing barbed microneedle cardiac patch was developed that improved mechanical support for infarcted hearts [55]. The design induced controlled micro-bleeding and coagulation during insertion, further enhancing adhesion strength. In rat models, the barbed microneedle patch adhered tightly to the epicardium without detachment for over 30 minutes, with spiral barbs providing secure fixation extending from the epicardium into the myocardium. Despite these advantages, microneedle-based systems still present safety concerns: insertion can cause surface damage, tissue scarring, and vascular injury [124, 157–159]. These risks highlight the importance of optimizing microneedle geometry, penetration depth, and material selection to balance firm fixation with long-term biocompatibility.

While prior sections highlight advances in adhesive materials and patch architectures, these developments have largely progressed in parallel, with few integrated approaches that address their combined performance under dynamic epicardial conditions.

## Synergistic Approaches and Future Directions

### Mucin–Auxetic Conceptual Framework

In the context of epicardial patch applications, auxetic structures offer a favorable combination of adhesion strength, mechanical adaptability, and tissue compatibility, demonstrate strong potentialfor dynamic cardiac environments. Literature reports indicate that auxetic patches can achieve adhesion strengths up to 130 kPa, exceeding reported values for multilayer patches (0.63–17.5 kPa) and microneedle patches (up to 70 kPa) [160, 161]. Their unique lattice architecture enables expansion in both longitudinal and transverse directions during cardiac cycles, sustaining tissue conformability and prolonged contact without detachment or delamination. This property is particularly critical on the epicardium, where high motion, complex curvature, and the presence of pericardial fluid often compromise conventional patch adhesion.

Auxetic designs also facilitate interfacial fluid drainage, an often overlooked but vital feature for epicardial applications. By incorporating hollow or porous geometries, these structures allow excess pericardial fluid to escape, reducing slippage and enhancing adhesion in wet physiological conditions. Furthermore, their anisotropic and synclastic mechanical behaviour enables seamless conformity to the heart’s curvature and contraction dynamics, reducing risks of tissue strain, crack initiation, or edge peeling.

**Fig 5.**
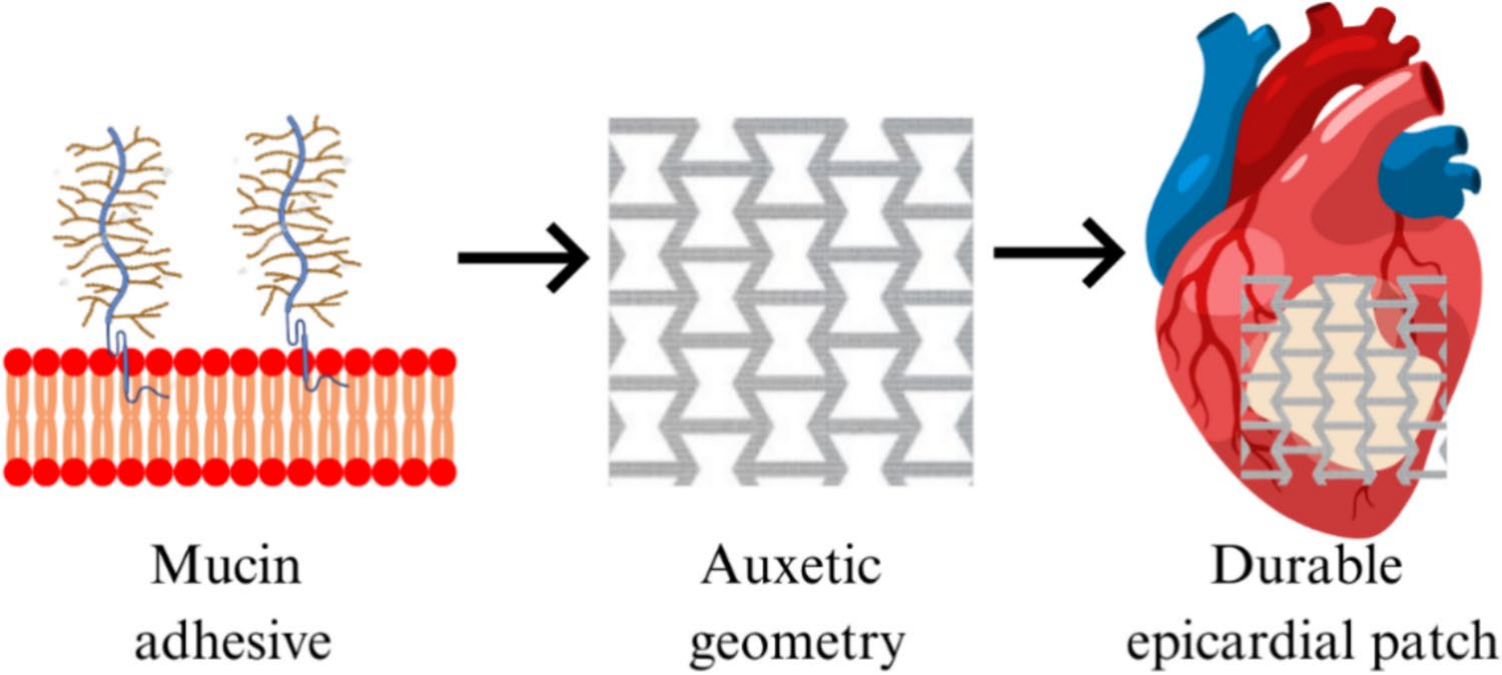
Conceptual framework illustrating the integration of mucin-based bioadhesion with auxetic structural geometry for the design of a durable epicardial patch

Mucin provides robust wet adhesion through hydration layers, dynamic bonding, and lubricity, while auxetic geometry ensures mechanical conformity, anisotropy, and synclastic adaptability under cyclic cardiac loading. When integrated, these features may yield a self-adhering, durable, and biocompatible epicardial patch capable of conformal contact with the heart surface. The feasibility of this integration is supported by prior demonstrations of auxetic cardiac patches (Fig 3b and c), which establish a mechanically adaptive scaffold suitable for coating or infiltration with mucin-based adhesive systems [28, 121]. This combined strategy as illustrated in Fig. 5, has the potential to advance epicardial repair and regeneration by simultaneously addressing the challenges of adhesion, mechanical resilience, and tissue compatibility.

Despite these advantages, auxetic patches face practical barriers to clinical translation. Large-scale manufacturing remains challenging, as achieving consistent lattice geometries with fine structural resolution is technically demanding. Sterilization methods such as gamma irradiation or autoclaving may alter material properties, compromising mechanical integrity and adhesion performance. In addition, the potential influence of auxetic scaffolds on native cardiac electrophysiology remains not fully characterised, raising concerns regarding arrhythmogenic risks. These limitations underscore the need for optimization of fabrication, sterilization, and functional validation before auxetic patches can be advanced to clinical use.

### Translational Challenges and Scalability 

In comparison, multilayer patches, although useful for controlled drug release or structural layering, face challenges on the epicardial surface due to thickness and stiffness mismatches, which may hinder adaptation to cyclic strain and  necessitate external adhesives or sutures for retention. Microneedle patches, while capable of physically anchoring into tissue, risk penetrating the myocardium, potentially inducing microtrauma, triggering inflammation, and increasing susceptibility to infection [124, 154, 162]. Additionally, their rigid barbs can fail or break during removal, limiting their clinical practicality. Overall, auxetic patches are positioned as a promising next generation epicardial interfaces, combining biomechanical conformity, stable adhesion, and minimal invasiveness, with the added benefit of being compatible with therapeutic delivery platforms [121, 163, 164]. The summary is shown in Table 2.

**Table 2.** Comparative analysis of auxetic vs conventional designs, highlighting on the adhesion strength, suitability, ease of fabrication and application, mechanical stability

Design / Properties	Auxetic structures	Multilayer patches	Microneedle patches
Adhesion strength (kPa)	Up to 130 [121]	0.63–17.5 [149]	Up to 70 [165]
Mechanical stability	Flexible, anisotropic, high strain tolerance, synclastic adaptability [30, 166]	Strong barrier against rupture; variable stability depending on layers [167, 168]	Stiffer and more brittle structure [157, 169]
Adhesion mechanism	Biomaterials form chemical bonds; lattice enables partial mechanical interlocking [124, 170]	Primarily dependent on chemical bonding of biomaterials [171, 172]	Physical anchoring via mechanical interlocking with epicardial tissue [54, 173]
Cardiac suitability	Adaptable, regulates interfacial fluid drainage through hollow lattice, reducing slippage [30]	Fabricated up to 4 mm for adhesion and regeneration; moderate fatigue resistance [160, 174]	Provides strong grip via penetration, but risks tissue damage and bacterial invasion [154, 173]
Ease of fabrication	Requires precision Molds or additive manufacturing to reproduce lattice geometry [128, 143]	Involves complex layer-by-layer casting and assembly [171, 172]	High risk of Mold/barb breakage during detachment or handling [54, 55, 175]
Ease of application	Requires bioadhesives for fixation [176, 177]	Thickness variation influences handling and placement [174]	Direct penetration of epicardium enables strong initial fixation [124, 154]

Values represent reported ranges or maximum adhesion strengths under experimental conditions in cited studies.kPa = kilopascal (10^3^ Pa). Synclastic deformation enables conformal contact with curved surfaces.

## Summary and Future Perspectives

This review provides a comprehensive perspective on biomimetic epicardial patches, highlighting recent advances in adhesive materials and structural designs aimed at overcoming the limitations of suturing and stapling. Bioadhesives such as mucin, dopamine, and chemically modified hyaluronic acid (HA), together with auxetic and multilayer architectures, have demonstrated the capacity to enhance adhesion and mechanical compliance under the dynamic cardiac  conditions of the heart. Among these, mucin remains relatively underexplored, despite its strong wet-tissue adhesion, while auxetic structures, characterized by a negative Poisson’s ratio, have shown superior conformability and mechanical stability without compromising tissue integrity. By contrast, dopamine-based adhesives, although effective in wet adhesion, remain hindered by oxidative instability and cytotoxic by-products.

A promising strategy lies in the integration of mucin-based adhesives with auxetic geometries. Mucin enables multivalent hydrogen bonding and hydrophobic interactions that replicate native mucus, while its lubricity reduces friction and mitigates inflammation. Auxetic lattices conform synclastically to epicardial curvature, evenly distributing stress and maintaining interfacial contact throughout cyclic cardiac motion. Together, these properties may enable the development of a self-adhering, conformal, and biocompatible epicardial patch that reduces reliance on sutures and supports long-term tissue integration. However, this mucin–auxetic synergy remains conceptual , requiring standardized testing protocols, reproducibility studies, and industrial scalability assessments before advancing toward clinical translation.

Despite these advantages, the clinical translation of mucin-based adhesives is still constrained by batch-to-batch variability, complex purification processes, and potential immunogenicity when derived from non-human sources. Addressing these challenges will require standardized production pipelines, scalable purification methods, and rigorous preclinical validation. Future research should focus on optimizing auxetic geometries to balance stiffness and anisotropy, and compliance, incorporating mucin with biocompatible polymers such as PEG derivatives, and conducting long-term in vivo studies under dynamic cardiac loading to ensure safety, durability, and functional integration.

## Data Availability

Not applicable. This is a review article based on previously published data.
